# Virus Inactivation Based on Optimal Surfactant Reservoir of Mesoporous Silica

**DOI:** 10.1021/acsabm.2c00901

**Published:** 2023-02-13

**Authors:** Rie Hirao, Keisuke Shigetoh, Shinji Inagaki, Nobuhiro Ishida

**Affiliations:** † Toyota Central R&D Labs., Inc., Nagakute, Aichi 480-1192, Japan

**Keywords:** virus inactivation, mesoporous silica, surfactant, micelle, wallpaper, aerosols

## Abstract

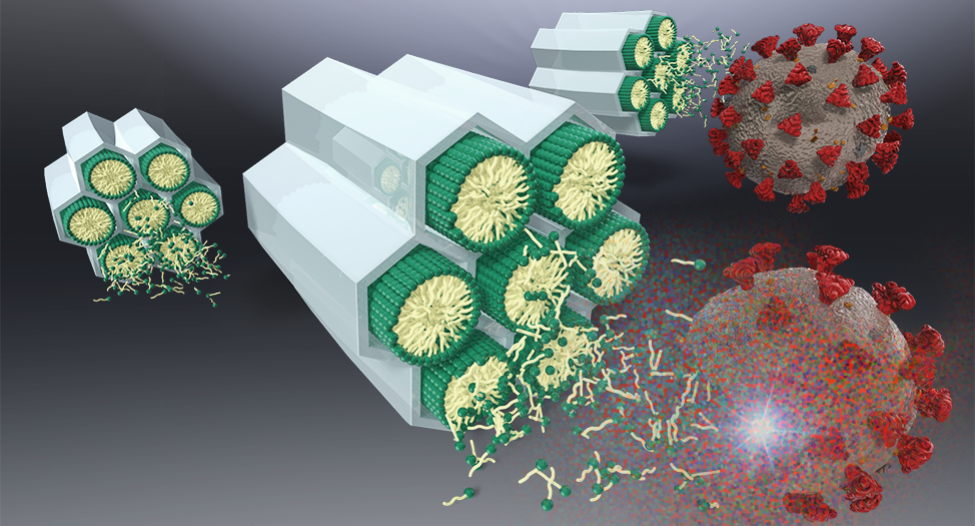

SARS-CoV-2 (severe acute respiratory syndrome coronavirus 2) caused a pandemic in 2019 and reaffirmed the importance of environmental sanitation. To prevent the spread of viral infections, we propose the application of a mesoporous silica (MS)-based virus-inactivating material. MS is typically synthesized using a micellar surfactant template; hence, the intermediate before removal of the surfactant template is expected to have a virus-inactivating activity. MS-CTAC particles filled with cetyltrimethylammonium chloride (CTAC), a cationic surfactant with an alkyl chain length of 16, were used to test this hypothesis. Plaque assays revealed that the MS-CTAC particles inactivated the enveloped bacteriophage φ6 by approximately 4 orders of magnitude after a contact time of 10 min. The particles also indicated a similar inactivation effect on the nonenveloped bacteriophage Qβ. In aqueous solution, CTAC loaded on MS-CTAC was released until the equilibrium concentration of loading and release on MS was reached. The released CTAC acted on viruses. Thus, MS is likely a good reservoir for the micellar surfactant. Surfactant readsorption also occurred in the MS particles, and the highest retention rate was observed when micellar surfactants with alkyl chain lengths appropriate for the pore size were used. The paper containing MS-CTAC particles was shown to maintain stable viral inactivation for at least three months in a typical indoor environment. Applying this concept to indoor wallpaper and air-conditioning filters could contribute to the inactivation of viruses in aerosols. These findings open possibilities for mesoporous materials with high surface areas, which can further develop into virus inactivation materials.

## 1 Introduction

The rapid and uncontrolled spread of SARS-CoV-2 (severe acute respiratory syndrome coronavirus 2) reported at the end of 2019 has caused severe damage to human health and economic activity worldwide.^1,2^ Infectious diseases caused by viruses have repeatedly occurred previously, including SARS, H1N1 influenza, and Middle East Respiratory syndrome (MERS), and there is concern that outbreaks may occur in the future.^3,4^ The most common viral infections are triggered by droplets or aerosols exhaled by an infected person.^5^ The virus in the aerosol eventually adheres to the surface of the material and maintains a stable infectivity for a certain period.^6^ SARS-CoV-2 analysis has reported that this virus retains infectivity on metal, plastic, cotton, and surgical mask surfaces for tens of hours to 7 days.^7,8^ Therefore, hygiene must be maintained in public spaces to prevent infection. Since the SARS-CoV-2 pandemic, public spaces have been subjected to regular and careful cleaning and ethanol disinfection, but this requires a great deal of effort. Moreover, some viruses are resistant to ethanol, such as norovirus.^9^ Sanitary materials with rapid and stable virus inactivation are required to prevent rapid pandemics in high-traffic and indoor spaces.

Viral particles typically have sizes of tens to hundreds of nanometers and are small compared to bacterial particles, which are on the micrometer scale. Viruses are classified based on the presence or absence of an envelope on the particle surface, and their surface structure and characteristics differ from those of bacteria. Furthermore, bacteria are single-celled living organisms, whereas virus depends on the host to multiply. Since bacteria and viruses differ greatly in size, structure, and growth process, the surfaces of virus-inactivating materials must be designed differently than those of antimicrobial properties.^10,11^ Recently, the development of materials targeting virus inactivation is advancing rapidly.^12^ In particular, many surface-coating materials utilizing silver, copper, and zinc oxide have been proposed,^13−17^ some of which are now in practical use. Moreover, the mechanisms of virus inactivation on material surfaces related to dissolved metal ions, reactive oxygen, and surface denaturation are being discussed.^18^ However, concerns have been raised on human toxicity and environmental impact caused by the rapid increase in functional materials.^12^ Material design appropriate for intended use is important, and various types of hygienic materials will be required in the future.

**Figure 1:**
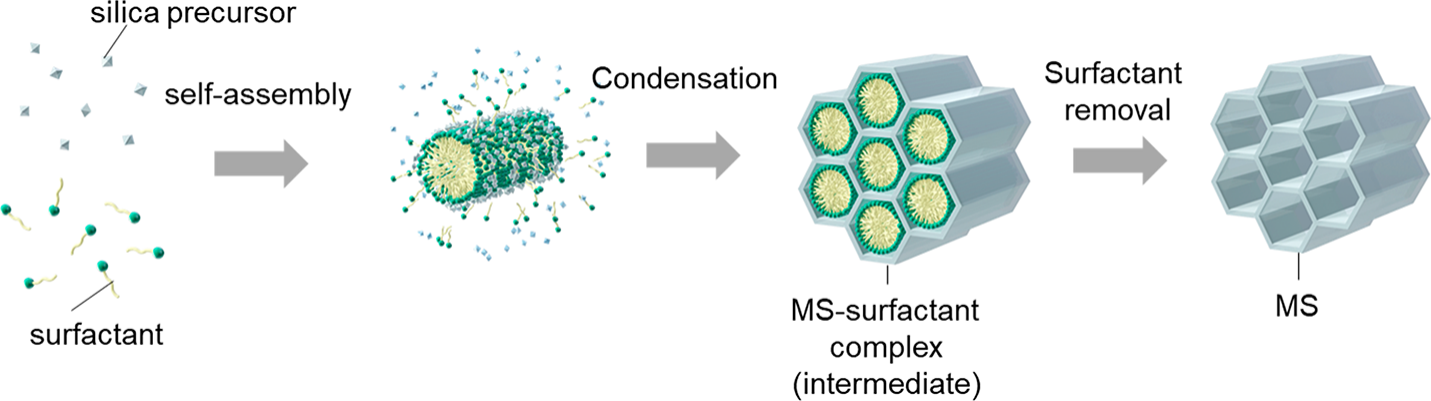
Schematic diagram of the production of MS-CTAC particles. Self-assembly and subsequent condensation of a silica precursor and surfactant produces an MS-surfactant complex (intermediate), and the surfactants are removed to obtain MS with a mesopore structure.

Mesoporous silica (MS) particles are known reservoir substrates for many antimicrobial materials. MS possesses a high surface area and loading capacity owing to its controlled pore structure, and its abundant chemical versatility allows for the control of the pore size, surface charge, and hierarchical structure.^19^ MS has been studied for catalyst and adsorbent applications, and since its initial report in the 1990s, various synthetic approaches have been developed and synthetic methods established, including new precursors, template design, hydrolysis conditions, and co-condensation and grafting techniques.^20,21^ MS particles are generally synthesized by hydrolysis and condensation of silica precursors around a template generated by supramolecular self-assembly (micellization) of a surfactant in an aqueous solution, followed by removal of the surfactant by calcination or solvent extraction (Figure 1). The unique structural features of MS have opened up numerous possibilities for fluorescent systems, sensors, charge transport materials, and solid catalysts as well as the immobilization of enzymes within MS pores to contribute to improved enzyme stability.^22^ The main ingredient, silicon dioxide (SiO_2_), is a Generally Recognized As Safe (GRAS)-classified material by the Food and Drug Administration (FDA), making it safe and biocompatible.^23^ Therefore, functional MS particles are expected to be used for drug delivery and as a fluorescent probe for nanoscale imaging.^24^ The pores in the MS serve as reservoirs to accommodate large quantities of small molecule compounds, biomolecules, and organic or inorganic imaging agents used in diagnostics.

Numerous types of antimicrobial MS have been reported to date, taking advantage of their high surface area and safety.^25,26^ Although MS particles themselves do not exhibit antimicrobial properties, it is possible to add antimicrobial properties by adsorbing or loading organic molecules (antibiotics, disinfectants, essential oils, fatty acids), metal nanoparticles, and metal complexes into the pores of MS particles. The antimicrobial efficacy of synthesized functional MS particles is generally significantly higher than that of free antimicrobial agents due to their localized loading effect.^27^ Vancomycin,^28^ ampicillin,^29^ chlorhexidine,^30^ and commercial biocide^31^ have been studied as antibiotics and fungicides to be loaded on MS, and their applications in films, hydrogels, fabrics, and bone implants have been proposed, along with analysis and control of adsorption and sustained release behavior. As for essential oils and fatty acids, MS particles loaded with vanillin,^32^ orange,^33^ cinnamon,^34^ lemon,^35^ and silylated natural fatty acids^36^ have been shown, and their use in biological devices is being considered due to the high biocompatibility of these loaders. In addition, there are numerous examples of MS particles loaded with Ag,^37^ Cu,^38,39^ and TiO_2_ nanoparticles,^40^ and factors such as the release of metal ions, disruption of cell membranes by the physical structure of the nanoparticles, and reactive oxygen species have been discussed as mechanisms causing antimicrobial function. Functional MS particles have been shown to have antimicrobial activity against Gram-positive bacteria (*Staphylococcus aureus*, *Bacillus subtilis*, *Enterococcus faecalis*, etc.) and Gram-negative bacteria (*Escherichia coli*, *Proteus mirabilis*, *Enterobacter aerogenes*, etc.),^25^ but the effectiveness of these materials against viruses is not explicit. Since silver and copper ions are originally known to have virus inactivation properties,^6,10^ some functional MS may be effective against viruses as well as bacteria.

**Figure 2:**
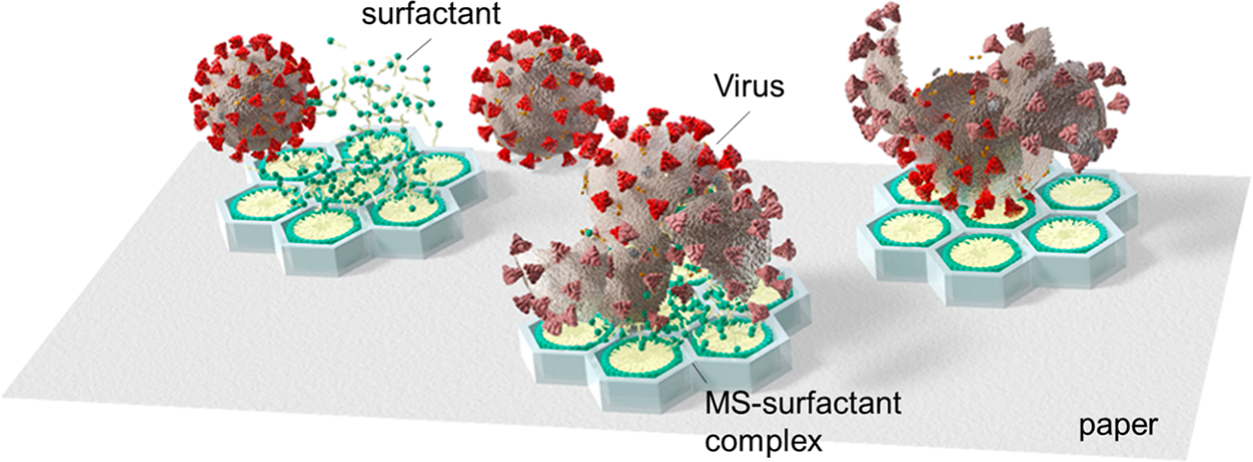
Conceptual diagram depicting how MS-CTAC inactivates viruses. The MS-surfactant complex is loaded onto paper, and the surfactant is released from the complex to inactivate the virus.

Here, we propose a virus inactivation material based on MS particles and its utilization to prevent the rapid spread of infectious diseases. The MS-surfactant complexes are expected to inactivate viruses. Among surfactants, quaternary ammonium salts are particularly effective against enveloped viruses, such as SARS-CoV-2.^41,42^ In this study, the bacteriophage φ6 as a model for enveloped viruses and bacteriophage Qβ as a model for nonenveloped viruses were selected to evaluate the virus inactivation potential of MS containing CTAC (MS-CTAC). Furthermore, a wallpaper containing MS-CTAC was proposed to prevent aerosol infection in indoor spaces (Figure 2). This application was evaluated for sustained virus inactivation over three months.

## 2 Experimental Section

### 2.1 Materials

The MS and MS-CTAC particles were obtained from Fuji Silysia Chemical (Aichi, Japan). MS-CTAC was synthesized from sodium silicate in the presence of cetyltrimethylammonium chloride (CTAC: C_16_H_33_N(CH_3_)_3_Cl) as a template. MS was prepared by calcination of MS-CTAC at 550 °C for 6 h in air. Silica gel (SG) particles (Fuji silica gel AB) were obtained from Fuji Silysia Chemical (Aichi, Japan). The N_2_ adsorption isotherms and textural properties of MS and SG are shown in Figure S1 and Table S1, respectively. CTAC, dodecyl trimethylammonium bromide (DTAB: C_12_H_25_N(CH_3_)_3_Br), and octadecyl methylammonium chloride (OTAC: C_18_H_37_N(CH_3_)_3_Cl) were obtained from Tokyo Chemical Industry (Tokyo, Japan), and their structures are provided in Figure S2.

### 2.2 Physicochemical Characterization of MS-CTAC

Scanning transmission electron microscopy (STEM) was performed using JEM-2100F (JEOL, Tokyo, Japan). The MS-CTAC particles were spotted on a grid (JEOL, Tokyo, Japan), and the dried samples were observed. Fourier transform infrared (FTIR) spectroscopy was performed using a Thermo Nicolet AVATAR 360 spectrophotometer (Thermo Fisher Scientific, Waltham, MA). Thermogravimetric analysis (TGA) was performed using a Thermo mass spectrometer (Rigaku, Tokyo, Japan). Using 5 mg of each adjusted MS particle, the percent change in the sample weight (TG%) and heat flow (μV) was measured. The temperature was increased at 20 °C min^–1^ in a He atmosphere, held at 100 °C for 25 min to volatilize moisture adsorbed on the powder, and then increased to 900 °C.

### 2.3 Viruses, Host Strains, and Media

Bacteria and viruses were obtained from the NITE Biological Resource Center (Chiba, Japan). Bacteriophage φ6 (NBRC 105899) was used as a model for enveloped viruses, and bacteriophage Qβ (NBRC 20012) was used as a model for nonenveloped viruses. The host strains used were *Escherichia coli* (NBRC 106373) and *Pseudomonas syringae* (NBRC 14084). LB (Luria–Bertani) medium (Formedium; Norfolk, UK) containing 2 mM calcium chloride (Ca-added LB medium) was used. The respective bacteriophages were infected after incubation at 37 °C for *E. coli* and 30 °C for *P. syringae* until the logarithmic growth phase. The culture plates were prepared by adding 1.5% (wt./vol.) agar powder (FUJIFILM Wako Pure Chemical, Osaka, Japan) to the Ca-added LB medium. Moreover, 0.6% (wt./vol.) agar powder was added to the Ca-added LB medium as the top agar for the plaque assay.

### 2.4 Viral Infection Titer

The viral infection titer was determined by plaque assay, a standard method for evaluating bacteriophages. A prepared sample of 500 μL of bacteriophage adjusted to a concentration of 1.3 × 10^6^ plaque forming units (PFU) in 1/500 NB (Nutrient broth) medium (Becton Dickinson, NJ) was added to 0.05 to 25 mg of each MS particle and shaken with a bioshaker (Deep well maximizer Bio Shaker M.BR-022UP, TAITEC, Tokyo, Japan) at 300 rpm at 25 °C. The interaction was then stopped by adding 1 mL of SCDLP (Soybean-Casein Digest with Lecithin & polysorbate 80) medium (Nihon Pharmaceutical, Osaka, Japan), and the supernatant after centrifugation (25 °C, 5000 rpm, 3 min) was used as a sample for the plaque assay. The contact time between the virus and MS particles was defined as the time between the virus addition and SCDLP medium addition. Because polysorbate 80 in SCDLP medium has been reported to inhibit the antimicrobial effect of quaternary ammonium salts,^43^ SCDLP medium was added to prevent further interaction between MS particles and the virus. Each solution was diluted with peptone-containing saline (Merck, Darmstadt, Germany), and 10 μL was added to 100 μL of the host culture medium in the log growth phase. After infection by standing at 37 °C and bacteriophage Qβ and standing at 25 °C for bacteriophage φ6 for 5 min, 4 mL of top agar was added and layered on bottom agar. After each bacterium was cultured, the number of plaques that appeared was measured using a colony counter Scan 500 (Interscience; Saint Nom la Bretêche, France). To elute the surfactant loaded in the MS pores, 20, 200, or 2000 mL of 1/500 NB medium was added to 200 mg of MS-CTAC particles, respectively, and the solution was allowed to stand for 30 min at room temperature. The solid material was collected and dried in a freeze-dryer for 3 h (VD-500R, TAITEC, Tokyo, Japan). Each sample before and after immersion in 1/500 NB medium was subjected to TGA.

### 2.5 Recycling MS-CTAC Particles

A prepared sample of 25 mg of MS-CTAC particles in 1 mL of 1/500 NB medium was vortexed for 10 s. After collecting the solid material by centrifugation (25 °C, 10 000 rpm, 1 min), another 1 mL of 1/500 NB medium was added to the precipitate and vortexed. This washing process was repeated 10 times. To the prepared powder, 2.1 × 10^6^ PFU of bacteriophage φ6 and 3.1 × 10^5^ PFU of bacteriophage Qβ were added and allowed to interact for 5 min, and the supernatant was collected by centrifugation (25 °C, 5000 rpm, 3 min) and used as a sample for the plaque assay.

### 2.6 Preparation of Surfactant-Adsorbed MS and SG Particles

When dissolved above a certain concentration, CTAC forms micelles in an aqueous solution with the hydrophilic groups facing outward, and the critical micelle concentration (CMC) is considered 1.58 mM.^44,45^ The CMC of OTAC is 0.33 mM,^46^ and the CMC of DTAB is 14 mM.^47^ To 4 mL of 100 mM surfactant (CTAC, OTAC, or DTAB) above the CMC, 100 mg of MS or SG particles was added to each solution and stirred for 1 h. After each sample was agitated and centrifuged at 10 000 rpm for 2 min, the solid material was collected and centrifuged with pure water to wash each MS particle. These samples were vacuum-lyophilized for 3 h (VD-500R; TAITEC, Tokyo, Japan). The surfactant adsorbed samples were named MS-reCTAC, MS-reDTAB, MS-reOTAC, and SG-reCTAC. As a control, a sample in which the equivalent of 1 mM CTAC below the CMC was readsorbed onto MS was also prepared using the same method and designated as MS-reCTAC-ucmc. We added 1 mL of pure water to 40 mg of each prepared MS particle sample, and the mixtures were allowed to stand at room temperature for 5 h. The solid material was collected and dried in a freeze-dryer for 3 h. Each treated sample was subjected to TGA.

### 2.7 Preparation of the Prototype Paper Containing MS-CTAC

The Kamino-moto kit (Awagami Factory, Tokushima, Japan) was used for traditional Japanese paper “washi” preparation. 5 g of recycled pulp (Chubu Electromagnetic Industry, Aichi, Japan) and 10 mL of 0.5 g L^–1^ viscosity agent were added to 300 mL of distilled water and stirred well. Half of the prepared solution (155 mL) was poured onto a 14.5 cm long and 10 cm wide duckboard to make the first pulp layer. For the remaining preparation, MS-CTAC (1 g) was dispersed in the remaining pulp solution, and the dispersion was added on the first pulp layer to yield double-layer pulp. The test paper was prepared by placing the weight on the double-layer pulp, removing water, and drying at 55 °C for 3 h. The MS-CTAC content in the test paper was approximately 7 mg cm^–2^. The detailed preparation method is presented in Figure S3. Scanning electron microscopy (SEM) analysis for of the prototype paper was performed using a TM3000 microscope (Hitachi High-Technologies, Tokyo, Japan) operated at 15 keV. MS-CTAC paper was spotted on carbon tape (JEOL, Tokyo, Japan). Prototype paper was cut into 1 cm^2^ and placed on 24 well plates with the MS-CTAC-loaded side up. Then, 120 μL of bacteriophage φ6, adjusted to a concentration of 1.0 × 10^7^ PFU mL^–1^ in 1/500 NB medium, was added dropwise, allowed to stand at room temperature for 30 min, and then placed in 5 mL tubes previously filled with 2 mL of SCDLP. The culture was then placed in a 5 mL tube containing 2 mL of SCDLP medium dispensed in advance. After light suspension, the tubes were centrifuged at 25 °C and 15 000 rpm for 1 min, and the supernatant was collected. The virus concentration in the supernatant was measured using the plaque assay method; three 1 cm^2^ sheets of paper were used to evaluate each sample, and the mean value was plotted.

## 3 Results and Discussion

### 3.1 Characterization of MS-CTAC Particles

**Figure 3:**
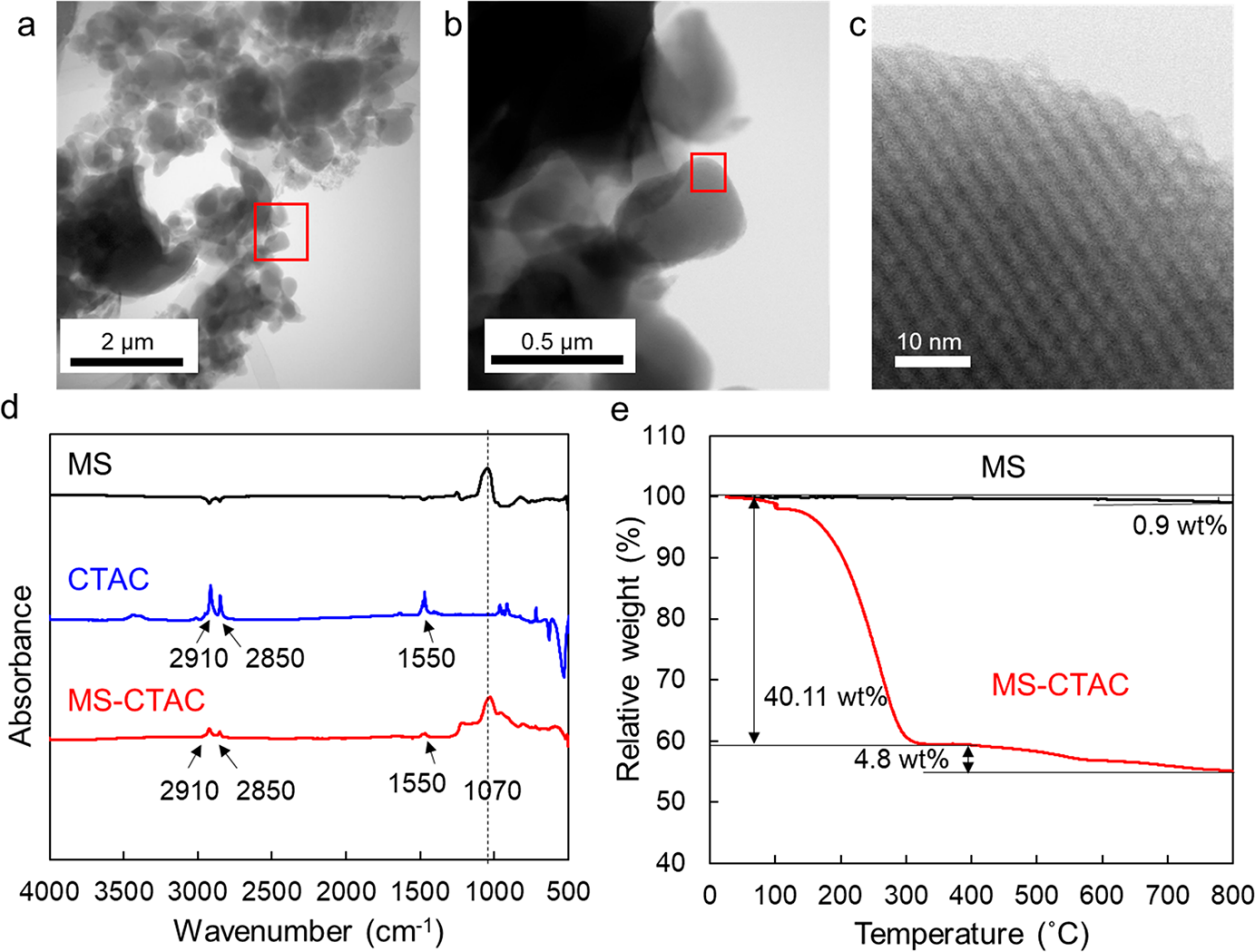
(a–c) BF-STEM images of MS-CTAC particles. The enlarged photograph of the red square area in (a) is shown in (b), and the enlarged photograph of the red square area in (b) is shown in (c). (d) FTIR spectra of MS particles, CTAC, and MS-CTAC particles. Arrows indicate spectral peaks characteristic of CTAC. Dotted lines indicate spectral peaks of MS. (e) TGA curve of MS and MS-CTAC particles. Shown as the relative change in weight of each particle before analysis.

MS-CTAC particles are an intermediate before removal of surfactant template obtained in the general synthesis process of MS particles (Figure 1). STEM, FTIR, and TGA were used to characterize this intermediate material. As depicted in Figure 3a,b, the bright-field (BF-) STEM image showed MS-CTAC particles as heterogeneous particles at the micrometer scale. The BF-STEM image also demonstrated a regular mesoporous structure of approximately 3.8 nm possessed by these particles (Figure 3c). The CTAC loading status was confirmed by FTIR analysis (Figure 3d). The MS and MS-CTAC particles showed absorption peaks, indicating Si–O asymmetric vibrations (1070 cm^–1^). As in a previous report,^48^ two absorption peaks (2850 and 2910 cm^–1^) attributed to the −CH_2_ stretching of the CTAC chain and one absorption peak (1550 cm^–1^) attributed to −CN stretching were observed only in the MS-CTAC particle.

Subsequently, TGA was used to determine the amount of CTAC loaded into the mesopores of the MS particles when they were heated to 900 °C under in He (Figure 3e). The MS particles as a control showed a very small decrease in relative weight of only 0.9 wt %. The small weight decrease is attributed to the dihydroxylation of surface silanol groups of MS. In general, silica NPs retain very high thermal stability and maintain a nearly constant weight at least up to 800 °C.^31^ However, MS-CTAC particles exhibited a significant weight loss of 40.11 wt % from 30 to 300 °C and a further weight loss of 4.8 wt % when the temperature reached 900 °C. Because the melting point of CTAC is 232–234 °C, this decrease is assumed to be due to CTAC pyrolysis. The weight ratio of CTAC loaded on MS-CTAC particles was 44.01 wt %, which is the final weight loss ratio in MS-CTAC minus 0.9 wt % of the weight loss in the MS particles. There was no visible evidence of residual coal, and CTAC is considered to be completely decomposed in He at 900 °C.

### 3.2 Virus Inactivation by MS-CTAC Particles

**Figure 4:**
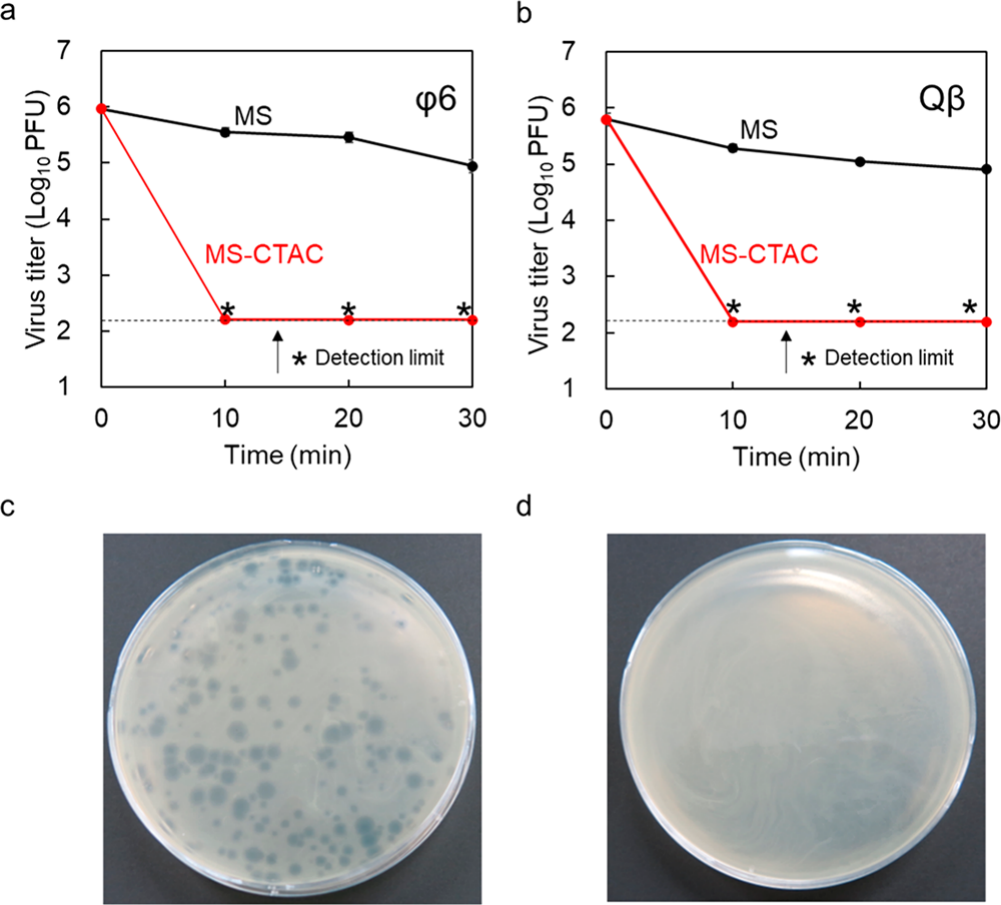
(a) Virus inactivation effects of MS-CTAC particles against bacteriophage φ6 of enveloped viruses and (b) bacteriophage Qβ of nonenveloped viruses. The red lines represent MS-CTAC, and the black lines represent MS. (c) Photographs of the plaque assay before MS-CTAC contact. (d) Photographs of the plaque assay after MS-CTAC contact.

The inactivation efficacy of MS-CTAC particles was evaluated using enveloped and nonenveloped viruses. For bacteriophage φ6 and bacteriophage Qβ with approximately 1.0 × 10^6^ PFU, 50 mg of MS-CTAC particles was added and shaken for up to 30 min. Then, the respective virus infection titer was determined by a plaque assay. As shown in Figure 4a,b, MS-CTAC particles demonstrated approximately 4 orders of magnitude of virus inactivation against their respective viruses. Quaternary amine surfactants such as CTAC exhibit inactivating effects against enveloped viruses.^49^ However, the MS-CTAC particles failed to reveal differences for both viruses because the particles showed inactivation below the detection limit against both viruses, even after only 10 min of contact time. The MS particles, which were CTAC-unloaded controls, showed a weak inactivation effect on either virus. A plate photograph of the plaque assay before contact with MS-CTAC (Figure 4c) and a plate photograph of the plaque assay after contact with MS-CTAC are shown (Figure 4d). The virus inactivation effect of MS-CTAC particles is suggested to be because of the loaded cationic surfactant. The mechanism of inactivation is thought to be mainly the progression of membrane disruption and protein denaturation caused by the interaction of the positive charge of the cationic surfactant with the lipid membrane and proteins that make up the outermost layer of the virus.^41,50^ As more than half of the SARS-CoV-2 inactivators approved by the U.S. Environmental Protection Agency (EPA) are quaternary ammonium salts,^51^ the use of surfactants as virus inactivators is considered an appropriate tool. The amount of MS-CTAC was reduced for comparison because the evaluation of 25 mg of MS-CTAC particles showed no difference in the virus inactivation effect on both viruses.

### 3.3 Recyclability of MS-CTAC Particles

**Figure 5:**
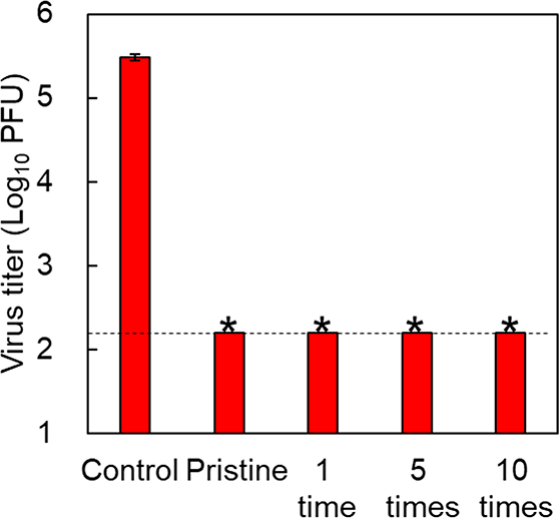
Recycling of MS-CTAC particles. The infection titer of bacteriophage φ6 in contact with MS-CTAC particles was washed up to 10 times. All examinations were performed in triplicate, independently, and results are presented as mean values with standard deviations. Asterisks (*) and the dotted black line indicate virus titer under the detection limit.

To test whether MS-CTAC particles are recyclable, 25 mg of MS-CTAC particles was thoroughly agitated in a 1 mL aqueous solution of 1/500 NB medium by vortexing, and the solid material was collected by centrifugation. This washing procedure was performed 10 times, and the virus inactivation effect of MS-CTAC particles was examined after 1, 5, and 10 washes using a plaque assay. Figure 5 shows the virus infection titer of the bacteriophage φ6 after 10 min of contact with each MS-CTAC particle. No plaque was detected even after washing the particles up to 10 times, confirming that MS-CTAC particles maintained their effect of virus inactivation against enveloped viruses. This examination was also performed on the bacteriophage Qβ, and the same effect was observed even for envelope-type viruses (Figure S4). MS-CTAC particles proved to be a stable reservoir material for recycled surfactants. However, the concentration of CTAC eluted varied with the number of MS-CTAC particles and the amount of water; therefore, our observation of the stability of MS-CTAC as a reservoir is limited to the conditions used in this experiment. Because the mesopores of MS are essentially formed using micellar CTAC as a template, CTAC is expected to be densely and stably adsorbed in the pores of the MS-CTAC particles.

### 3.4 Virus Inactivation by CTAC in Aqueous Solution

To determine the amount of CTAC eluted from MS-CTAC into aqueous solution, 200 mg of MS-CTAC particles was immersed in 20, 200, or 2000 mL of 1/500 NB medium for 30 min at room temperature. The particles were then separated and dried, and the residual loading amount of CTAC in each MS particle was determined using TGA. TGA (Figure S5) shows that the weight loss after 30 min of water immersion was approximately 1.67 wt %.

**Figure 6:**
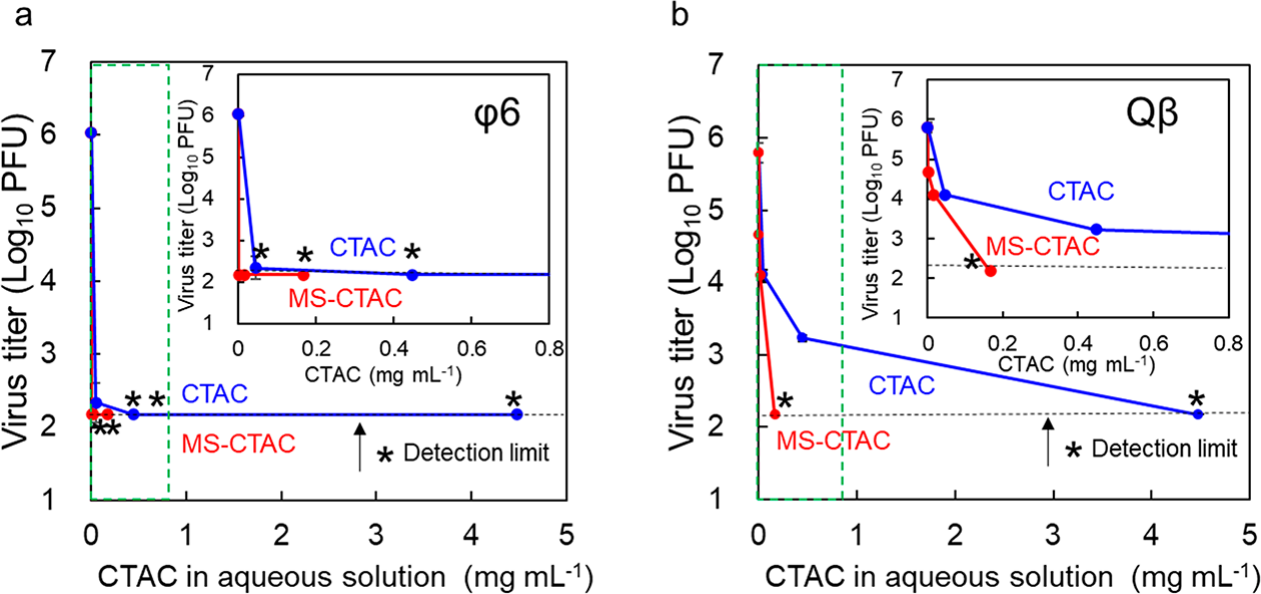
Virus inactivation effects of MS-CTAC particles and CTAC against bacteriophage φ6 of enveloped viruses (a) and bacteriophage Qβ of nonenveloped viruses (b). CTAC in aqueous solution (mg mL^–1^) on the horizontal axis of MS-CTAC is the CTAC concentration determined using TGA when MS-CTAC was immersed in 1/500 NB medium for 30 min. The red lines represent MS-CTAC, and the blue lines represent CTAC. The inset expands the area within the dashed green line, with the same *x*- and *y*-axis units. All examinations were performed independently in triplicate, and results are presented as mean and standard deviation. Asterisks (*) and the dotted black lines indicate virus titer under the detection limit.

Figure 6 shows a comparison of the virus inactivation effect between MS-CTAC and CTAC alone. The CTAC concentration (a horizontal axis) for MS-CTAC was calculated from the released amounts of CTAC into the solution. There was no difference in the antiviral effects between MS-CTAC and CTAC for bacteriophage φ6, as all were below the detection limit in the concentration range tested (Figure 6a). However, for bacteriophage Qβ, MS-CTAC exhibited higher inactivation by approximately 1 order of magnitude than CTACs at the same concentration (Figure 6b). The higher inactivation effect for MS-CTAC would be attributed to MS alone having also ab inactivation effect of virus with 1 order of magnitude after 30 min as shown in Figure 4.

### 3.5 Surfactant Adsorption by MS

**Figure 7:**
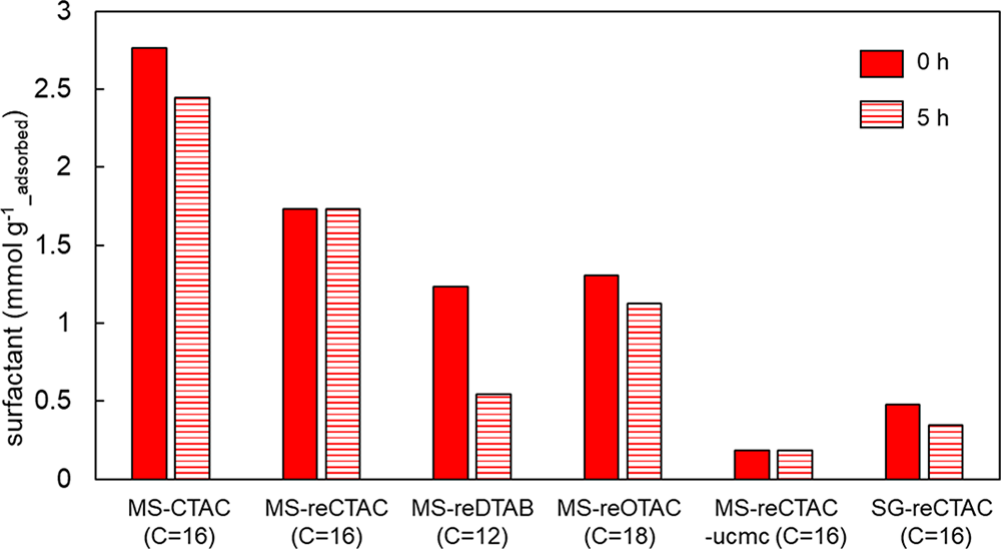
Number of moles of MS-CTAC or each surfactant readsorbed per gram of MS or SG particle initially (0 h) and after 5 h of immersion in aqueous solution (5 h), shown by TGA. The number of carbons in the alkyl chain of the surfactant is shown in parentheses next to the *x*-axis labels.

Three cationic surfactant (CTAC, OTAC, and DTAB) solutions prepared above the critical micelle concentration (CMC) were added to surfactant-free MS. The weight ratio of MS to CTAC in MS-CTAC was approximately 1:1 (Figure 3e); thus, the MS was mixed with CTAC in aqueous solution at approximately 1:2 (MS:CTAC weight ratio) to ensure an excess of surfactant in the adsorption test. Each MS with one of three surfactants (CTAC, DTAB, and OTAC) adsorbed above the CMC is denoted as MS-reCTAC, MS-reDTAB, and MS-reOTAC, respectively; MS with CTAC adsorbed below the CMC is denoted as MS-reCTAC-ucmc. Similarly, SG with CTAC adsorbed above the CMC is denoted as SG-reCTAC. The amount of each surfactant adsorbed in the MS was measured using TGA. As shown in Figures 7 and S7, the amount of CTAC adsorbed for MS-reCTAC was approximately 60% of the CTAC content of MS-CTAC. The molarity of each surfactant per adsorbed gram of MS particle was highest for CTAC, while DTAB and OTAC were both only approximately 70% adsorbed against CTAC. However, at 0.1 mM, which corresponds to less than the CMC, a very low amount of CTAC was adsorbed into the MS mesopore. SG adsorbed CTAC at much lower levels than that adsorbed on MS. Next, these surfactant-adsorbed particles were immersed in water for 5 h, and the degree to which each surfactant leached into the water was examined using TGA. Weight changes before and after immersion showed that MS-reCTAC was the most stable with almost no CTAC release. More than 60% of DTAB was eluted in the water, indicating that it was unstable in the MS pores of MS-reDTAB

Each surfactant was adsorbed into the MS pores in the micellar state. The micelle size depends on the alkyl chain length, with DTAB (C = 12), CTAC (C = 16), and OTAC (C = 18) being the largest in that order. This phenomenon is also supported by the fact that CTAC at less than the CMC, which did not form micelles, could not be adsorbed in the pores (MS-reCTAC-ucmc). DTAB and OTAC were inferior to CTAC; however, OTAC indicated a slight advantage in adsorbing and stability. This may be because the alkyl chain length of OTAC was closer to that of CTAC than that of DTAB. If DTAB or OTAC are used as surfactants in synthesizing mesoporous materials, they may be the most stable performers. This finding is important in various applications. Retreatment with surfactants above the micellar concentration allows the reuse of MS material with a weakened virus inactivation effect.

### 3.6 Virus Inactivation of MS-CTAC Containing Paper

**Figure 8:**
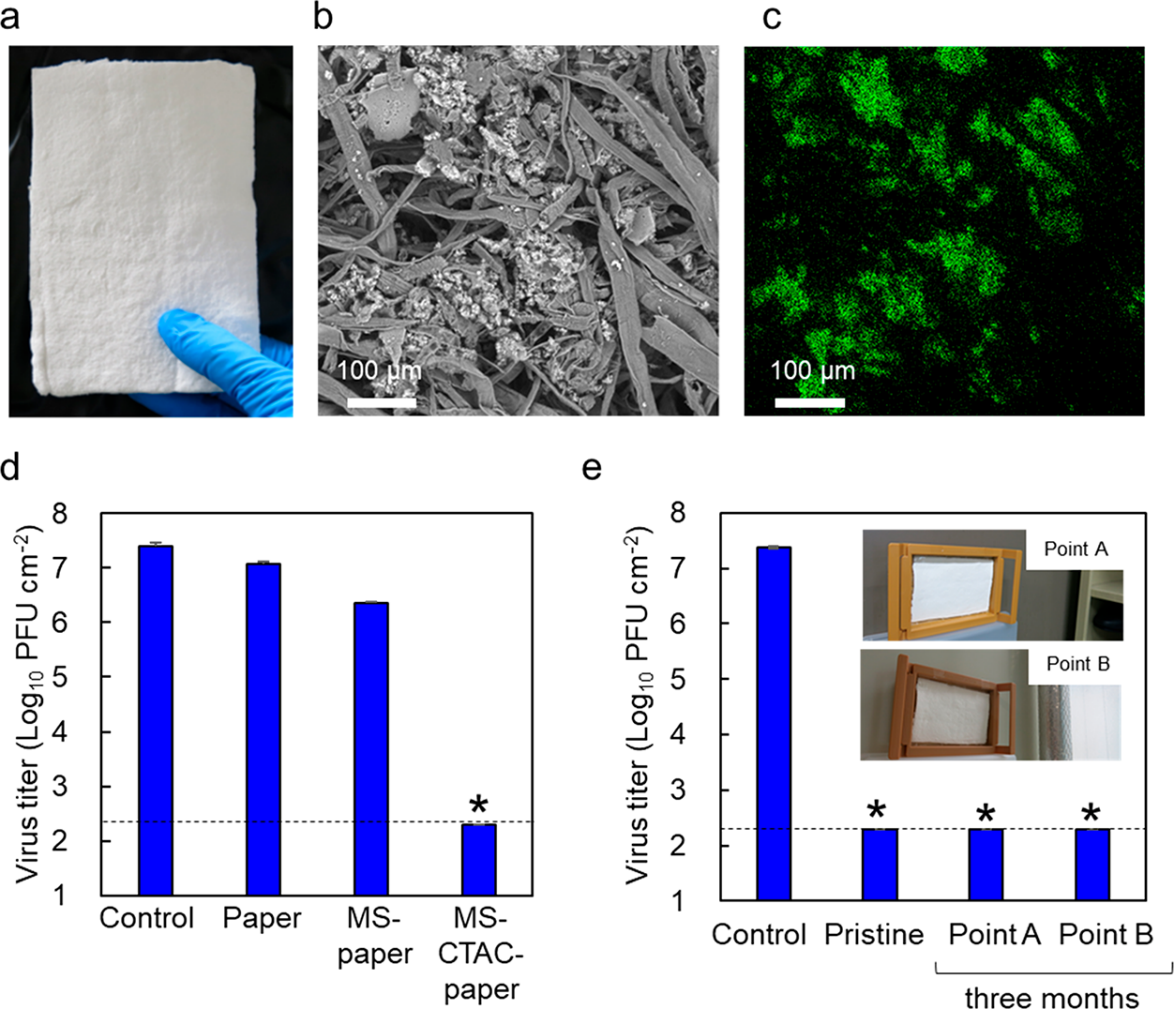
(a) Photograph of prepared paper loaded with MS-CTAC particles. (b) SEM image for the prepared paper. (c) Si mapping with EDX of SEM image of (b). (d) Virus inactivation effects of the prepared paper against bacteriophage φ6 viruses. (e) Viral infection titer against bacteriophage φ6 on prototype paper placed in the laboratory for three months including near the door (Point A) and the window (Point B). All examinations were performed in triplicate, independently, and presented as mean values and standard deviations. Asterisks (*) and dotted black lines indicate virus titer levels under the detection limit.

To develop an application of MS-CTAC particles, a prototype paper containing MS-CTAC particles was produced to evaluate the virus inactivation effect. The reason for focusing on paper was the expectation that this material could be utilized as indoor wallpaper or air filters as one possible means of preventing the dispersion of aerosols originating from droplets of infected people. The spread of the virus through aerosols has been identified as a cause of viral infections, highlighting the importance of air-conditioning. Viruses in aerosols are expected to be inactivated when they come in contact with wallpaper or air filters via indoor airflow control. The preparation of the paper containing MS-CTAC particles was based on the process of making Washi, a traditional Japanese paper (Figures 8a and S3). The prototype paper was confirmed to contain silica particles using SEM-EDX (Figures 8b and S7). If the particles were uniformly dispersed, 7.0 mg cm^–2^ of MS-CTAC particles would be contained in this paper. The virus infection titer is shown in Figure 8d, after 30 min of contact time with 7.4 log_10_ PFU of bacteriophage φ6 for 1 cm^2^ of this prototype paper. Consistent with previous findings, only the paper containing MS-CTAC particles demonstrated approximately a 5 orders of magnitude of virus inactivation. The two control samples, paper without particles and MS-particle-containing paper, also showed a slight decrease in the viral infection titer from 6.4 to 7.1 log_10_ PFU.

For use as indoor wallpaper, the prototype paper was placed in the laboratory for three months to evaluate the persistence of the virus inactivation effect. The prototype paper was installed in two locations: near doors, where people enter and exit (Point A), and near windows, where sunlight hits the paper (Point B). Figure 8e shows the viral infection titer of each sample (1 cm^2^) in contact with the bacteriophage φ6 for 30 min. After three months, the prototype paper showed approximately 5 orders of magnitude of virus inactivation at both locations.

The SARS-CoV-2-mediated pandemic has increased the need for sanitary products that retain virus inactivation performance. Measures to prevent contact and aerosol transmission are needed in public places where many people come and go. Despite periodic cleaning and ethanol disinfection being performed at various locations, it is still a labor-intensive process. Therefore, there is a need for highly sustainable virus-inactivating materials that do not require such work.^6^ However, some recommendations are concerning regarding the expected environmental impact of disposing of the rapidly growing number of quaternary amines and metals.^10,51^ Our proposed paper containing MS-CTAC particles is expected to be used for air-conditioning filters and wallpaper. Furthermore, the high retention of the surfactant and the ability to refill may reduce the amount of surfactant used. However, since this is an evaluation against a model virus, bacteriophage, revalidation against SARS-CoV-2 remains to be performed. The base material, mesoporous silica, has been approved as a safe material,^23^ and efforts are underway to try to apply it in medical applications.^24^ Films, hydrogels, polymers, and fabrics with antimicrobial MS have also been proposed.^25^ We expect that MS-CTAC particles can be deployed in various materials using a similar approach.

## 4 Conclusions

MS-CTAC particles, an MS intermediate, were shown to have an effective virus-inactivation effect. These particles proved to have a high inactivation effect, especially against enveloped viruses. The quaternary amines packed in the pores were stable in a micellar state, suggesting that MS is an excellent reservoir for surfactants. Since the prototype paper containing MS-CTAC particles proved superior in virus inactivation, it can be expected to inactivate viruses in aerosols when used in indoor wallpaper and air-conditioning filters. To the best of our knowledge, this is the first attempt to demonstrate MS intermediates as virus-inactivated materials. In addition to SARS-CoV-2, which caused the pandemic, the spread of infection by mutated or unknown viruses is a concern for the future. Research on sanitary materials with virus inactivation is less abundant than that on antimicrobial materials, and further development is desired in this field.

## Supporting Information Available

**Figure S1**. N_2_ adsorption isotherms of MS and SG. **Table S1**. Texture properties of MS and SG determined by N_2_ adsorption isotherms. **Figure S2**. Chemical structure of surfactants. **Figure S3**. Preparation of prototype paper with reference to the traditional Japanese paper Washi and cross-sectional view of prototype paper. **Figure S4**. Bacteriophage Qβ in contact with MS-CTAC particles was washed up to 10 times. **Figure S5**. TGA curve of MS-CTAC particles treated by immersion in 1/500 NB medium for 30 min. **Figure S6**. TGA curve of the elution of each surfactant readsorbed by the MS particles into water. **Figure S7**. SEM photomicrographs and EDX spectra of paper and MS-CTAC paper (PDF)

## References

[ref1] Li J.; Lai S.; Gao G. F.; Shi W. The Emergence, Genomic Diversity and Global Spread of SARS-CoV-2. Nature 2021, 600 (7889), 408–418. 10.1038/s41586-021-04188-6. 34880490

[ref2] Florindo H. F.; Kleiner R.; Vaskovich-Koubi D.; Acúrcio R. C.; Carreira B.; Yeini E.; Tiram G.; Liubomirski Y.; Satchi-Fainaro R. Immune-Mediated Approaches Against COVID-19. Nat. Nanotechnol. 2020, 15 (8), 630–645. 10.1038/s41565-020-0732-3. 32661375 PMC7355525

[ref3] Cui J.; Li F.; Shi Z.-L. Origin and Evolution of Pathogenic Coronaviruses. Nat. Rev. Microbiol. 2019, 17 (3), 181–192. 10.1038/s41579-018-0118-9. 30531947 PMC7097006

[ref4] Wu Z.; Harrich D.; Li Z.; Hu D.; Li D. The Unique Features of SARS-CoV-2 Transmission: Comparison with SARS-CoV, MERS-CoV and 2009 H1N1 Pandemic Influenza Virus. Rev. Med. Virol. 2021, 31 (2), e2171 10.1002/rmv.2171. 33350025 PMC7537046

[ref5] Wang C. C.; Prather K. A.; Sznitman J.; Jimenez J. L.; Lakdawala S. S.; Tufekci Z.; Marr L. C. Airborne Transmission of Respiratory Viruses. Science 2021, 373 (6558), eabd9149 10.1126/science.abd9149. 34446582 PMC8721651

[ref6] Sun Z.; Ostrikov K. Future Antiviral Surfaces: Lessons from COVID-19 Pandemic. Sustainable Mater.Technol. 2020, 25, e00203 10.1016/j.susmat.2020.e00203.

[ref7] Chin A. W. H.; Chu J. T. S.; Perera M. R. A.; Hui K. P. Y.; Yen H.-L.; Chan M. C. W.; Peiris M.; Poon L. L. M. Stability of SARS-CoV-2 in Different Environmental Conditions. Lancet Microbe. 2020, 1 (1), e10 10.1016/S2666-5247(20)30003-3. 32835322 PMC7214863

[ref8] van Doremalen N.; Bushmaker T.; Morris D. H.; Holbrook M. G.; Gamble A.; Williamson B. N.; Tamin A.; Harcourt J. L.; Thornburg N. J.; Gerber S. I.; Lloyd-Smith J. O.; de Wit E.; Munster V. J. Aerosol and Surface Stability of SARS-CoV-2 as Compared with SARS-CoV-1. N. Engl. J. Med. 2020, 382 (16), 1564–1567. 10.1056/NEJMc2004973. 32182409 PMC7121658

[ref9] Kampf G. Efficacy of Ethanol Against Viruses in Hand Disinfection. J. Hosp. Infect. 2018, 98 (4), 331–338. 10.1016/j.jhin.2017.08.025. 28882643 PMC7132458

[ref10] Rakowska P. D.; Tiddia M.; Faruqui N.; Bankier C.; Pei Y.; Pollard A. J.; Zhang J.; Gilmore I. S. Antiviral Surfaces and Coatings and Their Mechanisms of Action. Commun. Mater. 2021, 2 (1), 53 10.1038/s43246-021-00153-y.

[ref11] Bregnocchi A.; Jafari R.; Momen G. Design Strategies for Antiviral Coatings and Surfaces: A Review. Appl. Surf. Sci. Adv. 2022, 8, 100224 10.1016/j.apsadv.2022.100224.

[ref12] Imani S. M.; Ladouceur L.; Marshall T.; Maclachlan R.; Soleymani L.; Didar T. F. Antimicrobial Nanomaterials and Coatings: Current Mechanisms and Future Perspectives to Control the Spread of Viruses Including SARS-CoV-2. ACS Nano 2020, 14 (10), 12341–12369. 10.1021/acsnano.0c05937. 33034443

[ref13] Hosseini M.; Chin A. W. H.; Williams M. D.; Behzadinasab S.; Falkinham J. O. III; Poon L. L. M.; Ducker W. A. Transparent Anti-SARS-CoV-2 and Antibacterial Silver Oxide Coatings. ACS Appl. Mater. Interfaces 2022, 14 (7), 8718–8727. 10.1021/acsami.1c20872. 35138100 PMC8848512

[ref14] Merkl P.; Long S.; McInerney G. M.; Sotiriou G. A. Antiviral Activity of Silver, Copper Oxide and Zinc Oxide Nanoparticle Coatings against SARS-CoV-2. Nanomaterials 2021, 11 (5), 1312 10.3390/nano11051312. 34067553 PMC8155969

[ref15] Mosselhy D. A.; Kareinen L.; Kivistö I.; Aaltonen K.; Virtanen J.; Ge Y.; Sironen T. Copper-Silver Nanohybrids: SARS-CoV-2 Inhibitory Surfaces. Nanomaterials 2021, 11 (7), 1820 10.3390/nano11071820. 34361206 PMC8308209

[ref16] Behzadinasab S.; Chin A.; Hosseini M.; Poon L.; Ducker W. A. A Surface Coating that Rapidly Inactivates SARS-CoV-2. ACS Appl. Mater. Interfaces 2020, 12 (31), 34723–34727. 10.1021/acsami.0c11425. 32657566 PMC7385996

[ref17] Behzadinasab S.; Williams M. D.; Hosseini M.; Poon L. L. M.; Chin A. W. H.; Falkinham J. O. III; Ducker W. A. Transparent and Sprayable Surface Coatings that Kill Drug-Resistant Bacteria Within Minutes and Inactivate SARS-CoV-2 Virus. ACS Appl. Mater. Interfaces 2021, 13 (46), 54706–54714. 10.1021/acsami.1c15505. 34766745 PMC8609913

[ref18] Hosseini M.; Behzadinasab S.; Benmamoun Z.; Ducker W. A. The Viability of SARS-CoV-2 on Solid Surfaces. Curr. Opin. Colloid Interface Sci. 2021, 55, 101481 10.1016/j.cocis.2021.101481. 34149298 PMC8205552

[ref19] Tao Y.; Ju E.; Ren J.; Qu X. Bifunctionalized Mesoporous Silica-Supported Gold Nanoparticles: Intrinsic Oxidase and Peroxidase Catalytic Activities for Antibacterial Applications. Adv. Mater. 2015, 27 (6), 1097–1104. 10.1002/adma.201405105. 25655182

[ref20] Yanagisawa T.; Shimizu T.; Kuroda K.; Kato C. The Preparation of Alkyltrimethylammonium–Kanemite Complexes and Their Conversion to Microporous Materials. Bull. Chem. Soc. Jpn. 1990, 63 (4), 988–992. 10.1246/bcsj.63.988.

[ref21] Inagaki S.; Fukushima Y.; Kuroda K. Synthesis of Highly Ordered Mesoporous Materials from a Layered Polysilicate. J. Chem. Soc., Chem. Commun. 1993, 8, 680–682. 10.1039/c39930000680.

[ref22] Takahashi H.; Li B.; Sasaki T.; Miyazaki C.; Kajino T.; Inagaki S. Catalytic Activity in Organic Solvents and Stability of Immobilized Enzymes Depend on the Pore Size and Surface Characteristics of Mesoporous Silica. Chem. Mater. 2000, 12 (11), 3301–3305. 10.1021/cm000487a.

[ref23] Younes M.; Aggett P.; Aguilar F.; Crebelli R.; Dusemund B.; Filipic M.; Frutos M. J.; Galtier P.; Gott D.; Gundert-Remy U.; Kuhnle G. G.; Leblanc J.-C.; Lillegaard I. T.; Moldeus P.; Mortensen A.; Oskarsson A.; Stankovic I.; Waalkens-Berendsen I.; Woutersen R. A.; Wright M.; Boon P.; Chrysafidis D.; Gurtler R.; Mosesso P.; Parent-Massin D.; Tobback P.; Kovalkovicova N.; Rincon A. M.; Tard A.; Lambre C. Re-Evaluation of Silicon Dioxide (E 551) as a Food Additive. EFS2 2018, 16 (1), e05088 10.2903/j.efsa.2018.5088. PMC7009582 32625658

[ref24] Singh R. K.; Patel K. D.; Leong K. W.; Kim H.-W. Progress in Nanotheranostics Based on Mesoporous Silica Nanomaterial Platforms. ACS Appl. Mater. Interfaces 2017, 9 (12), 10309–10337. 10.1021/acsami.6b16505. 28274115

[ref25] Bernardos A.; Piacenza E.; Sancenón F.; Hamidi M.; Maleki A.; Turner R. J.; Martínez-Máñez R. Mesoporous Silica-Based Materials with Bactericidal Properties. Small 2019, 15 (24), 1900669 10.1002/smll.201900669. 31033214

[ref26] Kaya S.; Cresswell M.; Boccaccini A. R. Mesoporous Silica-Based Bioactive Glasses for Antibiotic-Free Antibacterial Applications. Mater. Sci. Eng., C 2018, 83, 99–107. 10.1016/j.msec.2017.11.003. 29208293

[ref27] Li L.-l.; Wang H. Antibacterial Agents: Enzyme-Coated Mesoporous Silica Nanoparticles as Efficient Antibacterial Agents In Vivo. Adv. Healthcare Mater. 2013, 2 (10), 1298–1298. 10.1002/adhm.201370050. 23526816

[ref28] Qi G.; Li L.; Yu F.; Wang H. Vancomycin-Modified Mesoporous Silica Nanoparticles for Selective Recognition and Killing of Pathogenic Gram-positive Bacteria Over Macrophage-Like Cells. ACS Appl. Mater. Interfaces 2013, 5 (21), 10874–10881. 10.1021/am403940d. 24131516

[ref29] Nairi V.; Medda L.; Monduzzi M.; Salis A. Adsorption and Release of Ampicillin Antibiotic from Ordered Mesoporous Silica. J. Colloid Interface Sci. 2017, 497, 217–225. 10.1016/j.jcis.2017.03.021. 28285049

[ref30] Zhang J. F.; Wu R.; Fan Y.; Liao S.; Wang Y.; Wen Z. T.; Xu X. Antibacterial Dental Composites with Chlorhexidine and Mesoporous Silica. J. Dent. Res. 2014, 93 (12), 1283–1289. 10.1177/0022034514555143. 25319365 PMC4237641

[ref31] Michailidis M.; Sorzabal-Bellido I.; Adamidou E. A.; Diaz-Fernandez Y. A.; Aveyard J.; Wengier R.; Grigoriev D.; Raval R.; Benayahu Y.; D’Sa R. A.; Shchukin D. Modified Mesoporous Silica Nanoparticles with a Dual Synergetic Antibacterial Effect. ACS Appl. Mater. Interfaces 2017, 9 (44), 38364–38372. 10.1021/acsami.7b14642. 29022348

[ref32] Ruiz-Rico M.; Pérez-Esteve É.; Bernardos A.; Sancenón F.; Martínez-Máñez R.; Marcos M. D.; Barat J. M. Enhanced Antimicrobial Activity of Essential Oil Components Immobilized on Silica Particles. Food Chem. 2017, 233, 228–236. 10.1016/j.foodchem.2017.04.118. 28530570

[ref33] Balaure P. C.; Boarca B.; Popescu R. C.; Savu D.; Trusca R.; Vasile B.; Grumezescu A. M.; Holban A. M.; Bolocan A.; Andronescu E. Bioactive Mesoporous Silica Nanostructures with Anti-Microbial and Anti-Biofilm Properties. Int. J. Pharm. 2017, 531 (1), 35–46. 10.1016/j.ijpharm.2017.08.062. 28797969

[ref34] Jobdeedamrong A.; Jenjob R.; Crespy D. Encapsulation and Release of Essential Oils in Functional Silica Nanocontainers. Langmuir: the ACS journal of surfaces and colloids 2018, 34 (44), 13235–13243. 10.1021/acs.langmuir.8b01652. 30300551

[ref35] Liu H.; Ni Y.; Hu J.; Jin Y.; Gu P.; Qiu H.; Chen K. Self-Healing and Antibacterial Essential Oil-Loaded Mesoporous Silica/Polyacrylate Hybrid Hydrogel for High-Performance Wearable Body-Strain Sensing. ACS Appl. Mater. Interfaces 2022, 14 (18), 21509–21520. 10.1021/acsami.2c03406. 35500100

[ref36] Pędziwiatr-Werbicka E.; Miłowska K.; Podlas M.; Marcinkowska M.; Ferenc M.; Brahmi Y.; Katir N.; Majoral J.-P.; Felczak A.; Boruszewska A.; Lisowska K.; Bryszewska M.; El Kadib A. Oleochemical-Tethered SBA-15-Type Silicates with Tunable Nanoscopic Order, Carboxylic Surface, and Hydrophobic Framework: Cellular Toxicity, Hemolysis, and Antibacterial Activity. Eur. J. Chem. 2014, 20 (31), 9596–9606. 10.1002/chem.201402583. 24958393

[ref37] Tian Y.; Qi J.; Zhang W.; Cai Q.; Jiang X. Facile, One-Pot Synthesis, and Antibacterial Activity of Mesoporous Silica Nanoparticles Decorated with Well-Dispersed Silver Nanoparticles. ACS Appl. Mater. Interfaces 2014, 6 (15), 12038–12045. 10.1021/am5026424. 25050635

[ref38] Díez B.; Roldán N.; Martín A.; Sotto A.; Perdigón-Melón J. A.; Arsuaga J.; Rosal R. Fouling and Biofouling Resistance of Metal-Doped Mesostructured Silica/Polyethersulfone Ultrafiltration Membranes. J. Membr. Sci. 2017, 526, 252–263. 10.1016/j.memsci.2016.12.051.

[ref39] Laskowski L.; Laskowska M.; Fijalkowski K.; Piech H.; Jelonkiewicz J.; Jaskulak M.; Gnatowski A.; Dulski M. New Class of Antimicrobial Agents: SBA-15 Silica Containing Anchored Copper Ions. J. Nanomater. 2017, 2017, 1287698 10.1155/2017/1287698.

[ref40] Cendrowski K. Mesoporous Silica Nanospheres Functionalized by TiO_2_ as a Photoactive Antibacterial Agent. J. Nanomed. Nanotechnol. 2013, 04, 182 10.4172/2157-7439.1000182.

[ref41] Schrank C. L.; Minbiole K. P. C.; Wuest W. M. Are Quaternary Ammonium Compounds, the Workhorse Disinfectants, Effective against Severe Acute Respiratory Syndrome-Coronavirus-2?. ACS Infect. Dis. 2020, 6 (7), 1553–1557. 10.1021/acsinfecdis.0c00265. 32412231 PMC10464937

[ref42] Ijaz M. K.; Nims R. W.; Zhou S. S.; Whitehead K.; Srinivasan V.; Kapes T.; Fanuel S.; Epstein J. H.; Daszak P.; Rubino J. R.; McKinney J. Microbicidal Actives with Virucidal Efficacy Against SARS-CoV-2 and Other Beta- and Alpha-coronaviruses and Implications for Future Emerging Coronaviruses and Other Enveloped Viruses. Sci. Rep. 2021, 11 (1), 5626 10.1038/s41598-021-84842-1. 33707476 PMC7952405

[ref43] Takasaki A.; Hashida T.; Kato K.; Nishihara T. Neutralizing Effect of Polysorbate on Bactericidal Action of A Quaternary Ammonium Disinfectant, Didecyldimethyl Ammonium Chloride, against Staphylococcus aureus. Eisei kagaku 1994, 40 (4), 351–356. 10.1248/jhs1956.40.351.

[ref44] Wang Z.; Larson R. G. Molecular Dynamics Simulations of Threadlike Cetyltrimethylammonium Chloride Micelles: Effects of Sodium Chloride and Sodium Salicylate salts. J. Phys. Chem. B 2009, 113 (42), 13697–13710. 10.1021/jp901576e. 19476369

[ref45] Tan C. H.; Huang Z. J.; Huang X. G. Rapid Determination of Surfactant Critical Micelle Concentration in Aqueous Solutions using Fiber-Optic Refractive Index Sensing. Anal. Biochem. 2010, 401 (1), 144–147. 10.1016/j.ab.2010.02.021. 20175982

[ref46] Kim H.-U.; Lim K.-H. Sizes and Structures of Micelles of Cationic Octadecyl Trimethyl Ammonium Chloride and Anionic Ammonium Dodecyl Sulfate Surfactants in Aqueous Solutions. Bull. Korean Chem. Soc. 2004, 25 (3), 382–388. 10.5012/bkcs.2004.25.3.382.

[ref47] Bahri M. A.; Hoebeke M.; Grammenos A.; Delanaye L.; Vandewalle N.; Seret A. Investigation of SDS, DTAB and CTAB Micelle Microviscosities by Electron Spin Resonance.. Colloids Surf., A 2006, 290 (1), 206–212. 10.1016/j.colsurfa.2006.05.021.

[ref48] Tang M.; Zhang P.; Liu J.; Long Y.; Cheng Y.; Zheng H. Cetyltrimethylammonium Chloride-Loaded Mesoporous Silica Nanoparticles as a Mitochondrion-Targeting Agent for Tumor Therapy. RSC Adv. 2020, 10 (29), 17050–17057. 10.1039/D0RA02023K. 35496920 PMC9053164

[ref49] Wood A.; Payne D. The Action of Three Antiseptics/Disinfectants Against Enveloped and Non-Enveloped Viruses. J. Hosp. Infect. 1998, 38 (4), 283–295. 10.1016/S0195-6701(98)90077-9. 9602977 PMC7134397

[ref50] McDonnell G.; Russell A. D. Antiseptics and Disinfectants: Activity, Action, and Resistance. Clin. Microbiol. Rev. 1999, 12 (1), 147–179. 10.1128/CMR.12.1.147. 9880479 PMC88911

[ref51] Hora P. I.; Pati S. G.; McNamara P. J.; Arnold W. A. Increased Use of Quaternary Ammonium Compounds During the SARS-CoV-2 Pandemic and Beyond: Consideration of Environmental Implications. Environ. Sci. Technol. Lett. 2020, 7 (9), 622–631. 10.1021/acs.estlett.0c00437. 37566314

