# Supplementary material for: Virus Inactivation Based on Optimal Surfactant Reservoir of Mesoporous Silica
Source: ACS Appl Bio Mater. 2023 Feb 13;6(3):1032–40. doi: 10.1021/acsabm.2c00901 (PMC10031556; doi:10.1021/acsabm.2c00901)
Supplement: Supplementary file 1 — mt2c00901_si_001.pdf [file mt2c00901_si_001.pdf]

Supporting information

Virus Inactivation Based on Optimal Surfactant Reservoir of Mesoporous Silica

Rie Hirao[a], Keisuke Shigetoh[a], Shinji Inagaki[a], Nobuhiro Ishida[a]*

[a] Toyota Central R&D Labs., Inc., 41-1 Nagakute, Aichi 480-1192, Japan

*E-mail: n-ishida@mosk.tytlabs.co.jp

a b

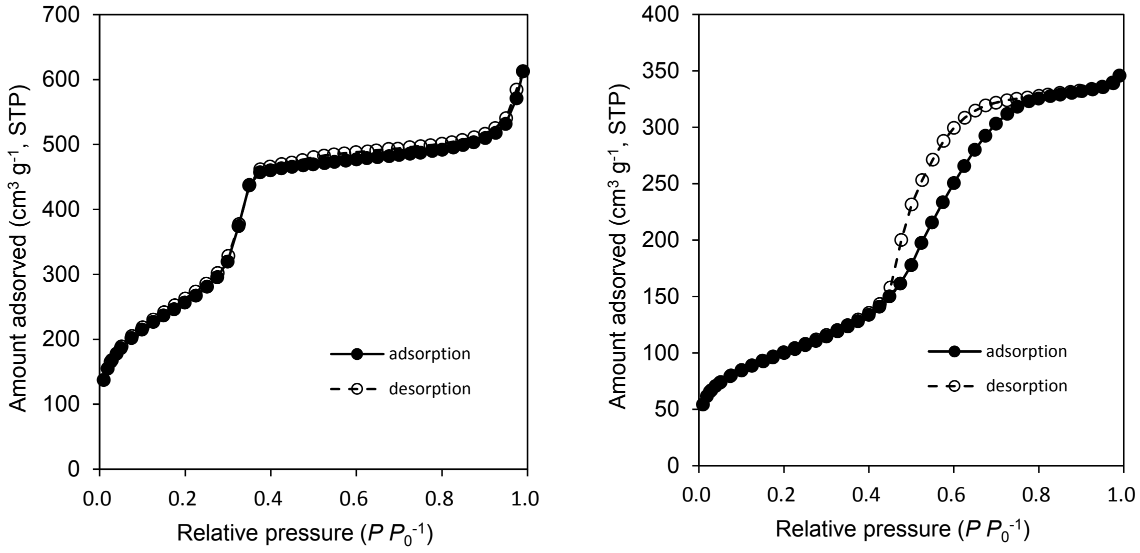

Figure S1. N_2 adsorption isotherms of MS (a) and SG (b). The isotherms were measured using a Quadrasorb (Quantachrome) at -196 ºC after outgassing at 100 ºC overnight.

Table S1. Texture properties of MS and SG determined by N_2 adsorption isotherms.

Sample	BET[a] surface area $m^2\ g^{-1}$	DFT[b] pore diameter nm	Pore volume[c] $cm^3\ g^{-1}$
MS	936	3.8	0.68
SG	361	5.6	0.47

[a] Brunauer-Emmett-Teller, [b] Density Functional Theory, [c] Determined by t-plot method.

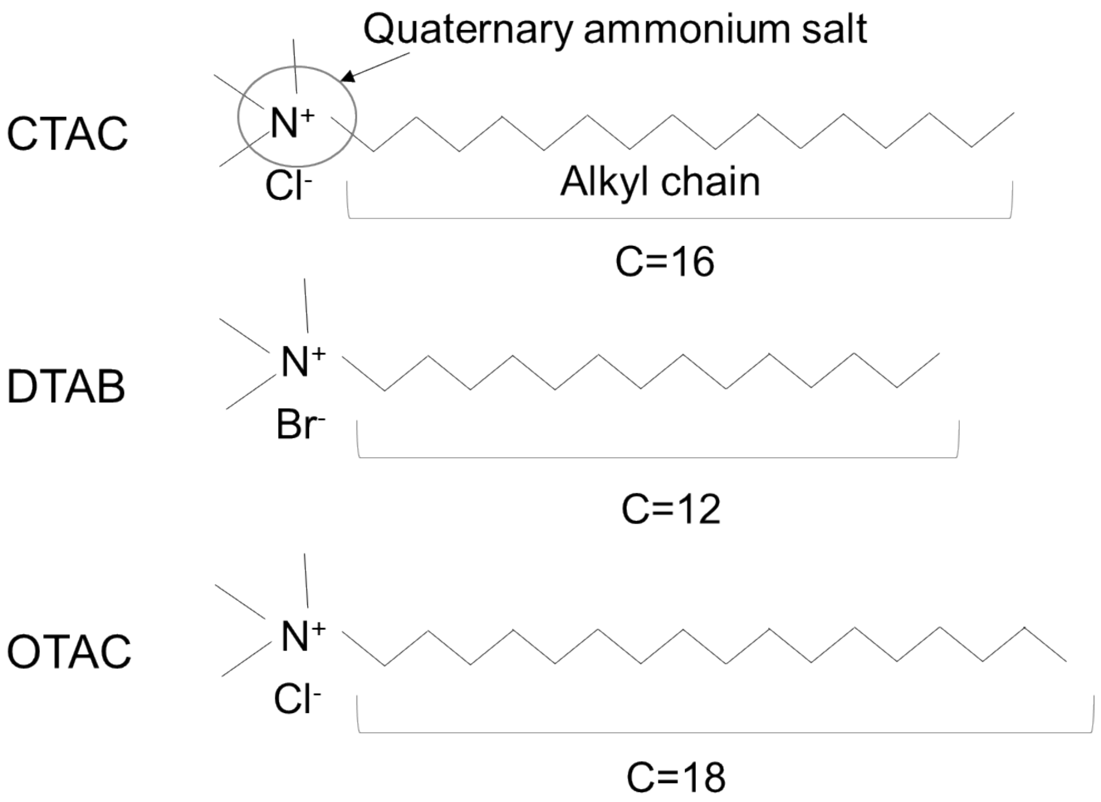

Figure S2. Chemical structure of surfactants.

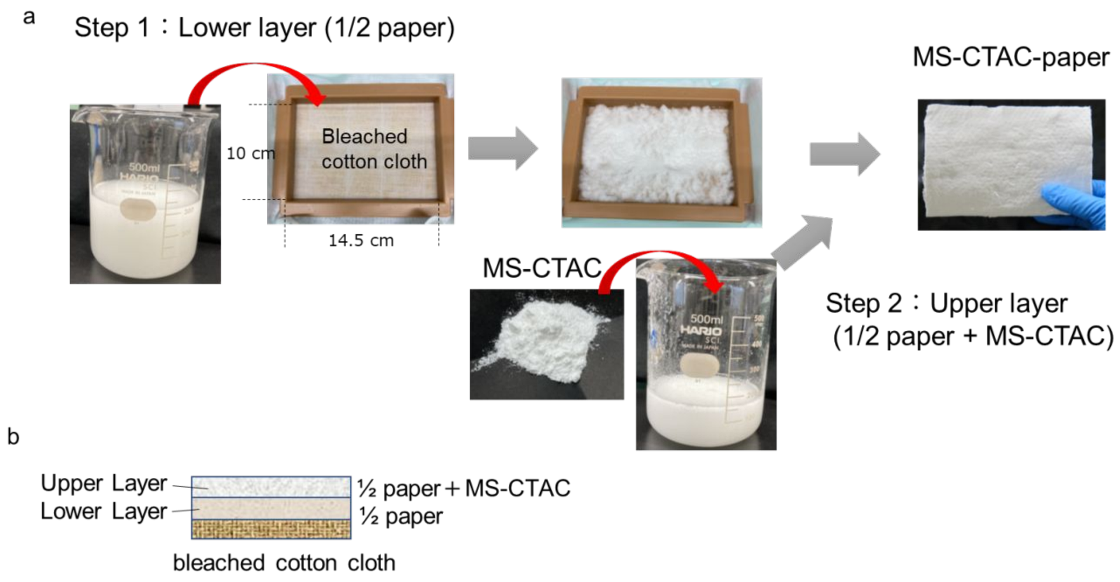

Figure S3. (a) Preparation of prototype paper with reference to the traditional Japanese paper Washi. (b) Cross-sectional view of prototype paper.

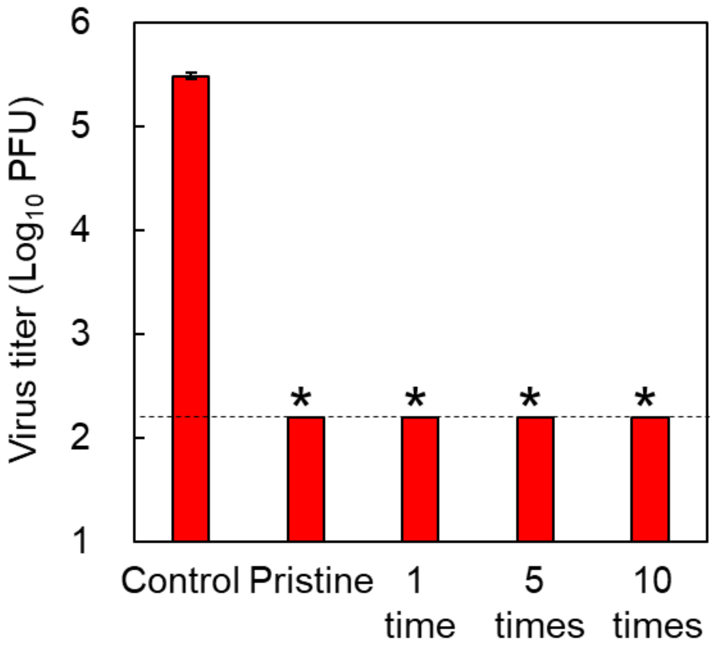

Figure S4. Virus titer of bacteriophage Qβ in contact with MS-CTAC particles after washing. Bacteriophage Qβ in contact with MS-CTAC particles was washed up to 10 times. All examinations were performed independently in triplicate, and results are presented as mean and standard deviation. Asterisks (*) and the dotted black line indicate virus titer under the detection limit.

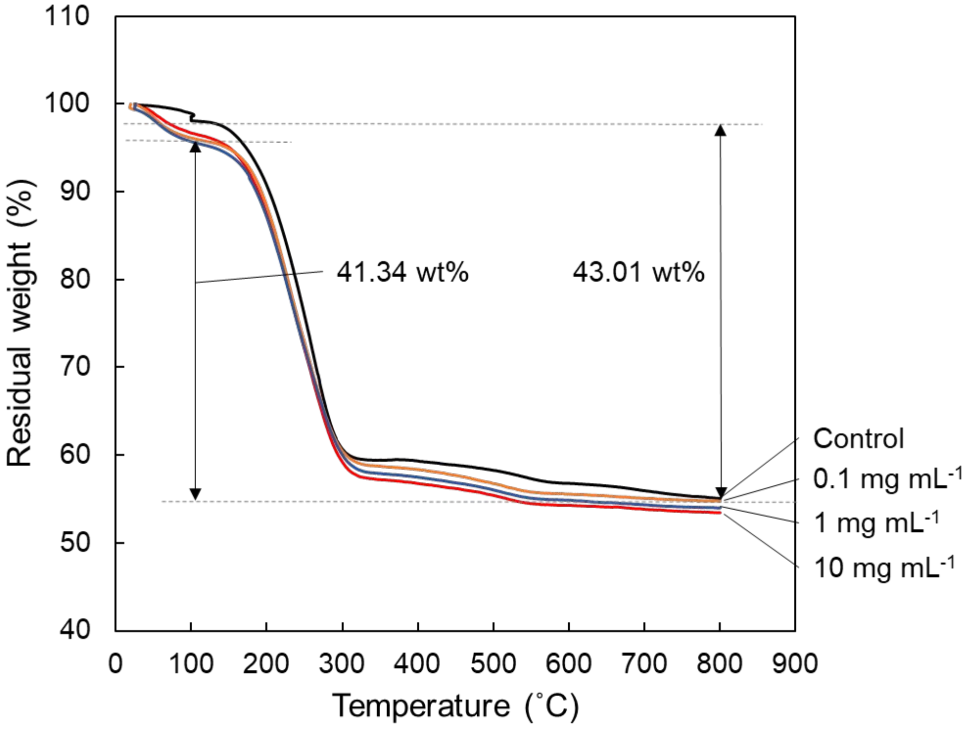

Figure S5. TGA curve of MS-CTAC particles treated by immersion in 1/500 NB medium for 30 min. 200 mg of MS-CTAC were immersed in 20, 200, and 2000 mL of 1/500 NB medium, respectively, for 30 min at room temperature (10 mg mL^{-1}, 1 mg mL^{-1}, 0.1 mg mL^{-1}). Control was MS-CTAC without immersion. The weight loss rate from 100°C to 800°C was compared between control and MS-CTAC dissolved in each volume of medium, and the weight of CTAC released into the medium was calculated. Excluded up to 100°C because it contains water absorption.

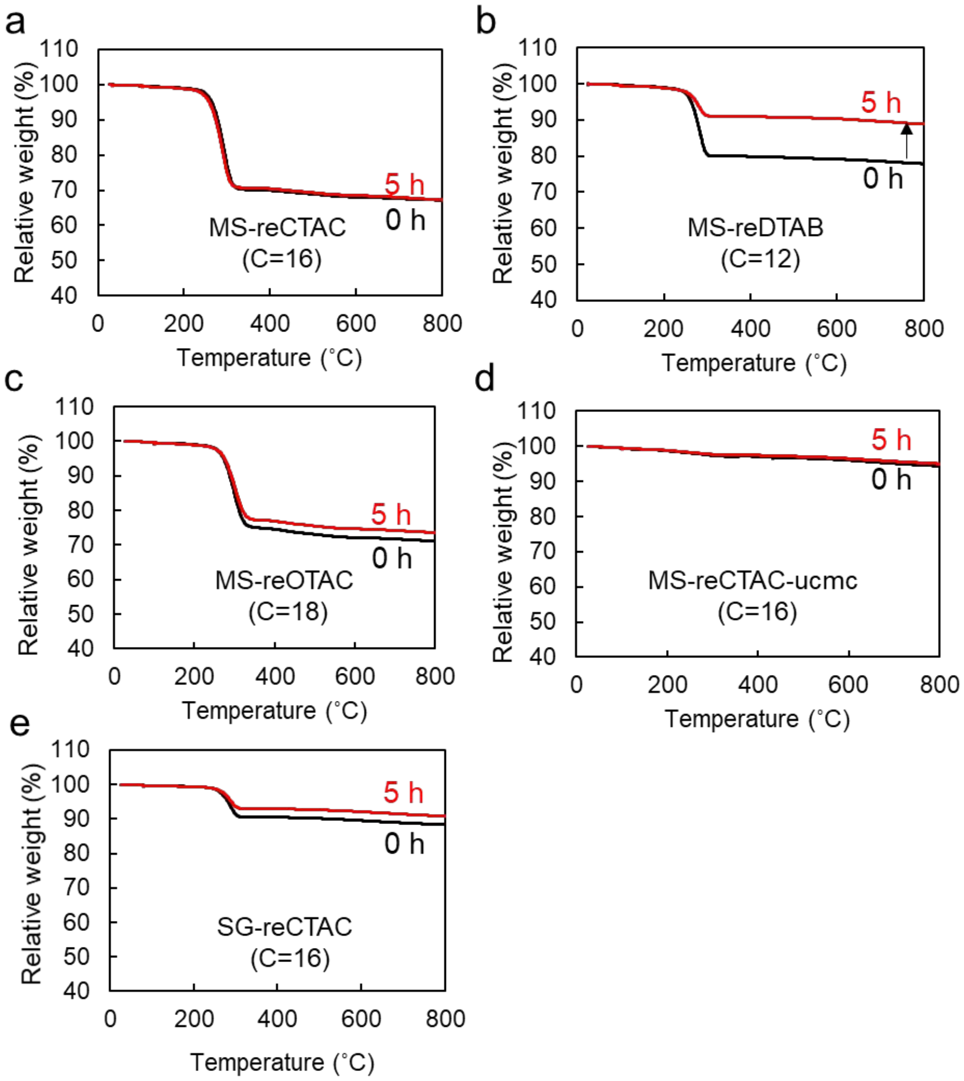

Figure S6. The MS with three surfactants (CTAC, DTAB, and OTAC) adsorbed above critical micelle concentration (CMC) is denoted as MS-reCTAC, MS-reDTAB, and MS-reOTAC, respectively. MS with CTAC adsorbed below CMC is denoted as MS-reCTAC-ucmc. Similarly, SG with CTAC adsorbed above CMC is labeled as SG-reCTAC. The graphs depict TGA curves of the elution of each surfactant reabsorbed by the MS particles into water for (a) MS-reCTAC, (b) MS-reDTAB, (c) MS-reOTAC, (d) MS-reCTAC-ucmc, and (e) SG-reCTAC. Parentheses contain the number of carbons in the alkyl chain of the surfactant.

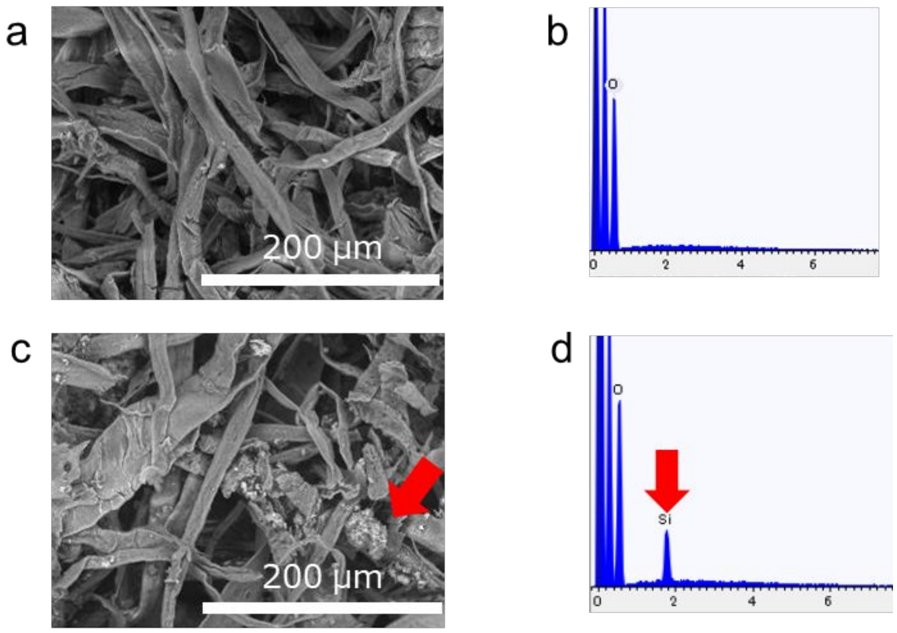

Figure S7. SEM photomicrographs and EDX spectra of paper (a and b) and MS-CTAC-paper (c and d). Red arrows indicate detected silicon (Si).
